# Differences in routine childhood immunization uptake between single and multiple healthcare facility use: the Kochi Adjunct Study of Japan Environment and Children’s Study

**DOI:** 10.1265/ehpm.25-00028

**Published:** 2025-06-28

**Authors:** Marina Minami, Yoshihiko Terauchi, Masamitsu Eitoku, Yuki Shimotake, Tamami Tsuzuki, Ryuhei Nagai, Nagamasa Maeda, Mikiya Fujieda, Narufumi Suganuma, The Kochi Adjunct Study of the Japan Environment and Children’s Study (JECS) Group

**Affiliations:** 1 Integrated Center for Advanced Medical Technologies (ICAM-Tech), Kochi Medical School Hospital, Nankoku, Kochi 783-8505, Japan; 2 Department of Pediatrics, Kochi Medical School, Kochi University, Nankoku, Kochi 783-8505, Japan; 3 Department of Environmental Medicine, Kochi Medical School, Kochi University, Nankoku, Kochi 783-8505, Japan; 4 Faculty of Nursing, Tohto University Numazu Campus, 1-1 Hinodecho, Numazu, Shizuoka 410-0032, Japan; 5 Department of Obstetrics and Gynecology, Kochi Medical School, Kochi University, Nankoku, Kochi 783-8505, Japan

**Keywords:** Immunization, Vaccination uptake, Pediatric clinic, Maternal and Child Health Handbook, JECS

## Abstract

**Background:**

The efficacy of routine childhood immunization depends on timely vaccine uptake and facility use patterns. This study examined the association between pediatric vaccination facility use patterns and routine childhood immunization uptake among children up to age eight years.

**Methods:**

As part of the Kochi Adjunct Study of the Japan Environment and Children’s Study (JECS), we analyzed data from 1,644 participants whose Maternal and Child Health Handbook photographs were collected in the eighth year of the cohort study. Maternal and Child Health Handbook records determined immunization completion. Participants were categorized into four groups based on pediatric vaccination facility use patterns: single facility use throughout, multiple facility use during the first period, multiple facility use during the second period, and multiple facility use throughout both periods. Maternal and child characteristics were collected via paper-based questionnaires. Associations between facility use patterns, sociodemographic factors, and immunization completion were analyzed using chi-square tests and logistic regression.

**Results:**

Overall, routine childhood immunization completion was observed in 1,259 (76.6%) participants. Chi-square tests indicated that marital status, educational level, lower parity, never smoking, not attending nursery, and breastfeeding practice for infants aged four months old were significantly associated with routine childhood immunization completion. Single facility use throughout the immunization period was observed in 1,011 (61.5%) participants. Multiple facility use (38.5%) was associated with higher odds of routine childhood immunization incompletion than single facility use. This association was the strongest for those who used multiple facilities throughout the vaccination period (adjusted odds ratio, 1.90; 95% confidence interval, 1.24–2.91).

**Conclusions:**

Single pediatric facility use was associated with higher routine immunization uptake. Our findings suggest that encouraging the use of one medical institution for a child’s vaccinations may be a useful approach to consider when addressing vaccination coverage challenges.

**Supplementary information:**

The online version contains supplementary material available at https://doi.org/10.1265/ehpm.25-00028.

## Graphical Abstract

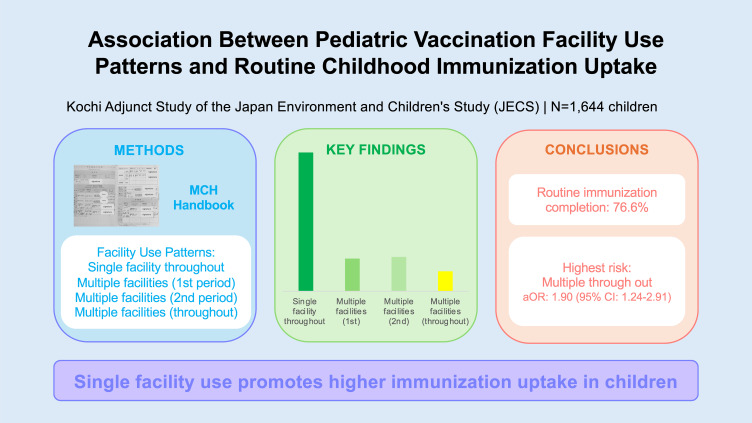

## 1. Background

Childhood vaccination, including primary immunization, is extensively considered a major preventive measure within the context of healthcare strategies for improving the population’s health and well-being. Beyond preventing diseases, vaccination provides significant economic and social benefits to individuals and society [1, 2]. Specifically, childhood vaccination reduces healthcare costs, prevents productivity losses, and improves children’s cognitive development, educational outcomes, health equity, and social integration [2, 3]. According to the World Health Organization, the ongoing efforts to improve global childhood vaccination coverage help prevent life-threatening diseases and 3.5–5 million deaths among children every year worldwide; however, this is only possible when vaccines reach the intended target population. The World Health Organization has set targets to achieve a childhood vaccination rate of 95% by 2030 [4].

Recently, the Japanese immunization program has made considerable progress. In addition to continuing to implement traditional primary routine immunization (i.e., inactivated polio vaccines, diphtheria, tetanus toxoid, and pertussis), several new vaccines (e.g., *Haemophilus influenzae* type b vaccine, pneumococcal conjugate vaccines, rotavirus vaccine, human papillomavirus vaccine, and cell-derived Japanese encephalitis vaccine) have been introduced in the Japan National Immunization Program [5]. This development of the National Immunization Program closes the vaccination gap in Japan relative to that of other high-income countries. Moreover, the immunization schedule for infants and young children in Japan has become more complicated owing to individual vaccination and an increase in the numbers and types of vaccinations [6].

Regardless of the progress, this routine childhood immunization schedule is very complex in terms of timing, type, and frequency [7, 8]. Previous studies examining immunization behavior and individual- and community-level factors associated with appropriately-timed infant immunization found that numerous factors—namely, not having a family physician during infancy, young age of mothers, late birth order, economic hardship, and maternal employment—were correlated with inadequate immunization coverage for multiple vaccinations [9]. Other studies also summarized the influencing factors of routine vaccination uptake, with some factors being immunization programs/services, logistics, health infrastructure, the perception of adverse effects, knowledge, beliefs, hesitancies, social influences, and other general parental and family attitudes toward children [10, 11]. Furthermore, studies analyzing risk factors for low vaccination rates in low- and middle-income countries have identified the following factors as relevant: low income, lack of access to healthcare, low parental education, and unassisted childbirth [12–14]. All these factors can lead to increased/decreased childhood vaccination uptake. There is also the situation that while some reports have highlighted the acceptance of multiple vaccines and an increase in the number of injectable vaccines when children go in a single immunization visit [15], detailed information—for both low- and middle-income countries and high-income countries—regarding whether routine childhood immunization uptake is associated differently with single and multiple healthcare facility use is lacking.

This study examined the association between pediatric vaccination facility use patterns and routine childhood immunization uptake among children aged up to eight years in Kochi Prefecture. Conducted as part of the Kochi Adjunct Study of the Japan Environment and Children’s Study (JECS), the primary outcome was routine childhood immunization completion according to the Maternal and Child Health Handbook (MCHH) records.

## 2. Methods

### 2.1. Study design and participants

This study was conducted as an Adjunct Study of the JECS by the Ministry of the Environment, Japan. The study protocol and baseline data of the JECS have been reported elsewhere [16, 17]. The JECS is an ongoing, nationwide, birth cohort study including over 100,000 pregnant women recruited between January 2011 and March 2014 and enrolled in 15 JECS study areas, which in turn cover a wide geographic area in Japan. For this study, 1,705 participants who presented at the eight-year follow-up of the JECS in Kochi Prefecture were initially enrolled for the Kochi Adjunct Study of the JECS.

This study was based on the JECS datasets jecs-ta-20190930 (2022.11.29ver) released in October 2019. The dataset used in this study covers the period from early pregnancy to three years of age, from which relevant data were extracted for this research. Data collection was conducted through paper questionnaires administered to mothers at three key time points: early pregnancy, late pregnancy, and during the one-month health check-up. Subsequent data collection occurred via mailed paper questionnaires distributed every six months. As part of the JECS, participants underwent physical measurements and developmental assessments when the children reached the age of eight years. During these in-person assessments, images of the MCHHs were obtained. The vaccination status data from these MCHH images were then combined with the questionnaire data collected by the JECS for analysis.

We examined vaccination patterns at two key time points, as follows: the first period when multiple vaccinations are needed (2–8 months old in Japan Pediatric Society recommendation) and the second period when multiple vaccinations are needed (12–24 months old in Japan Pediatric Society recommendation). These age strata were selected to reflect distinct immunological phases recognized in pediatric care, with the 2–8 month window representing a critical period for primary immunization series initiation, and the 12–24 month phase marking the transition to booster administration and expanded environmental exposures. The first period typically includes the primary series of Hib vaccine, pneumococcal vaccine, DTaP–IPV combination vaccine, and the BCG vaccination. The second period is characterized by booster shots for Hib, pneumococcal, and DTaP–IPV vaccines, as well as the primary dose of the MR vaccine. The enrolled participants were categorized into four groups based on their pediatric vaccination facility use patterns, as follows: single facility use throughout the immunization period, multiple facility use at the first period, multiple facility use at the second period, and multiple facility use throughout both periods of the immunization schedule.

### 2.2. Outcome variables

The primary outcome of this study was routine childhood immunization completion according to the MCHH records of children up to the age of eight years. The image records were verified by pediatricians to determine vaccination timing and the pediatric vaccination facility used. Furthermore, pediatric vaccination facility use during the first period and the second period and use pattern (single or multiple pediatric facilities) were determined using the MCHH records.

### 2.3. Maternal and Child Health Handbook

The MCHH in Japan (in Japanese, “*Boshi Techo*”), a comprehensive record-keeping tool for children’s health that allows the creation of detailed immunization records and ensures recording consistency from early pregnancy through childhood (i.e., it should contain detailed immunization records of children), was introduced in 1948 and has been a pioneering tool in safeguarding maternal and child health and welfare at the national level. Japan became the first country in the world to create and distribute such a comprehensive health record [18].

The data used in this study were extracted from MCHHs. Trained research assistants initially entered information from MCHH photos before a systematic verification by a board-certified pediatric infectious disease specialist, who confirmed the vaccination records, attending vaccination facility (single or multiple facilities), the name of the responsible pediatrician, and vaccination dates using standardized validation criteria, including recording consistency, date accuracy, and vaccine type confirmation (Additional File 1 for an example of an MCHH).

### 2.4. Other variables

Data on maternal characteristics, including age, educational level, marital status, parity, smoking habits, and employment status, were obtained through paper-based questionnaires in the first, second, and third trimesters. Participants completed all questionnaires twice during pregnancy (once in early pregnancy and once in late pregnancy) and once at a one-month postpartum check-up; subsequently, they received questionnaires sent by postal mail every six months.

Maternal age at delivery was categorized into six groups: <20, 20–24, 25–29, 30–34, 35–39, and ≥40 years. Maternal educational level was categorized into three groups, which were high school or less, vocational school/college, and university or higher. Marital status was categorized into married or single. Parity was categorized as first-time parents, one child, two or more children, while smoking habits were categorized into never smoked, quit smoking (before or after becoming pregnant), and current smoker. Employment status was categorized into working or non-employed/student. The characteristics of children, including parity, attending nursery at six months and one year old, and breastfeeding status at the age of four months old, were obtained through the paper-based questionnaires that the mothers completed when their children reached the ages of six months and one year old.

### 2.5. Statistical analyses

All results are expressed as numbers and percentages of the participating cases. The participants were divided into two groups based on routine childhood immunization completion, and a chi-square test was conducted to examine the basic characteristics of mothers and children. Subsequently, the participants were categorized into four groups based on pediatric vaccination facility use patterns, as follows: single facility use throughout the immunization period, multiple facility use during the first period, multiple facility use during the second period, and multiple facility use throughout both periods of the immunization schedule. Chi-square tests were then conducted to analyze the difference between these groups. To examine the association between routine childhood vaccination completion and pediatric vaccination facility use patterns and identify independent factors related to vaccination uptake, we conducted both crude and adjusted logistic regression analyses. In the adjusted analyses, we included variables that were significantly associated with vaccination completion in our preliminary analyses as well as factors previously identified in the literature as potentially influential. These adjustment variables encompassed sociodemographic characteristics, health-related factors, and healthcare utilization patterns. We performed all analyses using Stata/MP software (version 16.1; StataCorp). Post hoc power calculations were conducted using G*Power version 3.1. The primary analyses had 80% power to detect odds ratios of 1.5 or greater for binary outcomes, assuming a two-tailed test with α = 0.05. However, the subgroup analyses involving facility-use patterns (n = 4 strata) should be interpreted with caution, as we cannot rule out the potential for inflated Type II error arising from the multiple comparisons.

## 3. Results

**Fig. 1:**
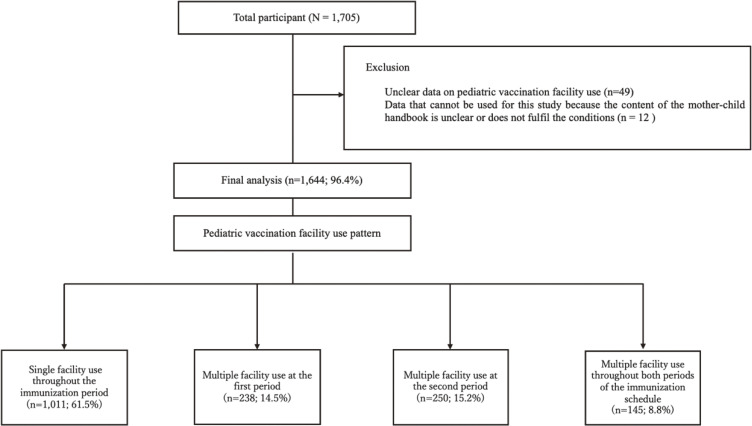
Flowchart showing the distribution of participants. The figure shows a flow diagram of participant selection and classification by pediatric vaccination facility use pattern. A total of 1,705 participants were initially assessed. After excluding cases with unclear or incomplete data, 1,644 were included in the final analysis. Participants were categorized into four groups based on their vaccination facility use during the routine childhood immunization schedule.

In total, 1,705 participants were initially considered for this study, among whom 49 were excluded owing to unclear data on pediatric vaccination facility use at 1–7 months old and between 1 and 2 years old, and 12 participants were excluded because the content of their MCHH was unclear or did not meet the study criteria. The final analysis included 1,644 cases, representing 96.4% of the initial sample (Fig. 1). The distribution of pediatric vaccination facility use patterns was as follows: single facility use throughout the immunization period (n = 1,011; 61.5%), multiple facility use during the first period (n = 238; 14.5%), multiple facility use during the second period (n = 250; 15.2%), and multiple facility use throughout both periods of the immunization schedule (n = 145; 8.8%; Fig. 1).

Table 1 presents the detailed demographic profiles of the 1,644 participants, with 76.6% completing routine childhood immunization. Several characteristics were significantly associated with completion (*p* < 0.05), including higher educational levels, married status, never-smoking status, first-time parenthood, non-attendance at nursery, breastfeeding at four months, and single facility use throughout the immunization period. Specifically, participants with vocational school/college and university education or higher had the highest completion rates, while married participants, never-smokers, and first-time parents showed higher completion rates compared to their counterparts. Those not attending nursery and those practicing breastfeeding at four months also had higher completion rates. Single facility use throughout the immunization period was associated with the highest completion rate compared to multiple facility use. Maternal age at delivery and employment status were not significantly associated with immunization completion.

**Table 1:** Participant demographic characteristics

		**Routine childhood immunization**		
	**All**	**Completion**	**Incompletion**	
	**1,644**	**1,259 (76.6)**	**385 (23.4)**	
	**n (%)**			***P***
Maternal age at delivery (years)				0.40
<20	7 (0.4)	6 (0.5)	1 (0.3)	
20–24	109 (6.6)	87 (6.9)	22 (5.7)	
25–29	458 (27.9)	363 (28.8)	95 (24.7)	
30–34	596 (36.3)	452 (35.9)	144 (37.4)	
35–39	402 (24.5)	300 (23.8)	102 (26.5)	
≥40	72 (4.4)	51 (4.1)	21 (5.5)	
Educational level				<0.05
High school or less	398 (24.2)	285 (22.6)	113 (29.4)	
Vocational school/college	759 (46.2)	583 (46.3)	176 (45.7)	
University or higher	477 (29.0)	383 (30.4)	94 (24.4)	
Missing	10 (0.6)	8 (0.6)	2 (0.5)	
Marital status				<0.05
Married	1,564 (95.1)	1,209 (96.0)	355 (92.2)	
Single	65 (4.0)	42 (3.3)	23 (6.0)	
Missing	15 (0.9)	8 (0.6)	7 (1.8)	
Smoking				<0.05
Never smoker	1,120 (68.1)	886 (70.4)	234 (60.8)	
Quit	467 (28.4)	339 (26.9)	128 (33.3)	
Current smoker	49 (3.0)	29 (2.3)	20 (5.2)	
Missing	8 (0.5)	5 (0.4)	3 (0.8)	
Employment status				0.07
Non-employed/student	567 (34.5)	445 (35.4)	122 (31.7)	
Work	1,023 (62.2)	779 (61.9)	244 (63.4)	
Missing	54 (3.3)	35 (2.8)	19 (4.9)	
Parity				<0.05
First-time parents	717 (43.6)	619 (49.2)	98 (25.5)	
One child	590 (35.9)	424 (33.7)	166 (43.1)	
Two or more children	268 (16.3)	154 (12.2)	114 (29.6)	
Missing	69 (4.2)	62 (4.9)	7 (1.8)	
Nursery attendance				<0.05
No	963 (58.6)	762 (60.5)	201 (52.2)	
At six months and one year	92 (5.6)	61 (4.9)	31 (8.1)	
At one year only	528 (32.1)	400 (31.8)	128 (33.3)	
At six months only	5 (0.3)	4 (0.3)	1 (0.3)	
Missing	56 (3.4)	32 (2.5)	24 (6.2)	
Breastfeeding at four months old				<0.05
No	216 (13.1)	157 (12.5)	59 (15.3)	
Yes	1,402 (85.3)	1,088 (86.4)	314 (81.6)	
Missing	26 (1.6)	14 (1.1)	12 (3.1)	
Pediatric vaccination facility use				0.05
Single facility use throughout	1,011 (61.5)	794 (63.1)	217 (56.4)	
Multiple facility use at the first period	238 (14.5)	178 (14.1)	60 (15.6)	
Multiple facility use in the second period	250 (15.2)	187 (14.9)	63 (16.4)	
Multiple facilities used throughout	145 (8.8)	100 (7.9)	45 (11.7)	

Participant demographic characteristics are stratified by routine childhood immunization completion. Values are presented as number and percentage (%). P-values indicate group differences assessed using chi-square tests.

Table 2 presents the distribution of pediatric vaccination facility use patterns among the 1,644 participants. Most (61.5%) used a single facility throughout the immunization period, while others used multiple facilities at different stages. Maternal age at delivery and educational levels were similar across facility use patterns (*p* = 0.41 and *p* = 0.45, respectively). Marital status showed a modest significant difference (*p* < 0.05), with married individuals being the majority in all categories. Smoking status varied significantly (*p* < 0.05), with never-smokers being the majority in all categories. Parity showed a trend toward significant difference (*p* = 0.06) among categories. Nursery utilization and breastfeeding at four months old did not differ significantly (*p* = 0.80 and *p* = 0.68, respectively). A modest significance (*p* = 0.05) was observed in the association between routine childhood immunization completion and single facility use throughout the immunization period (78.5%) compared to multiple the facility use categories.

**Table 2:** Distribution of pediatric vaccination facility use patterns during the routine childhood immunization period

		**Pediatric clinics**				

	**All**	**Single facility used throughout ** **the immunization period**	**Multiple facility ** **use at the first period**	**Multiple facilities used in the second period**	**Multiple facilities used throughout ** **the immunization ** **period**	
**n (%)**	**1,644**	**1,011 (61.5)**	**238 (14.5)**	**250 (15.2)**	**145 (8.8)**	
		
						***P***
Maternal age at delivery (years)						0.41
<20	7 (0.4)	5 (0.5)	1 (0.4)	1 (0.4)	0 (0.0)	
20–24	109 (6.6)	63 (6.2)	15 (6.3)	23 (9.2)	8 (5.5)	
25–29	458 (27.9)	269 (26.6)	79 (33.2)	60 (24.0)	50 (34.5)	
30–34	596 (36.3)	367 (36.3)	79 (33.2)	96 (38.4)	54 (37.2)	
35–39	402 (24.5)	258 (25.5)	57 (24.0)	60 (24.0)	27 (18.6)	
≥40	72 (4.4)	49 (4.9)	7 (2.9)	10 (4.0)	6 (4.1)	
Educational level						0.45
High school or less	398 (24.2)	247 (24.4)	61 (25.6)	59 (23.6)	31 (21.4)	
Vocational school/college	759 (46.2)	471 (46.6)	103 (43.3)	107 (42.8)	78 (53.8)	
University or higher	477 (29.0)	288 (28.5)	71 (29.8)	82 (32.8)	36 (24.8)	
Missing	10 (0.6)	5 (0.5)	3 (1.3)	2 (0.8)	0 (0.0)	
Marital status						<0.05
Married	1,564 (95.1)	970 (95.9)	224 (94.1)	237 (94.8)	133 (91.7)	
Single	65 (4.0)	34 (3.4)	9 (3.8)	13 (5.2)	9 (6.2)	
Missing	15 (0.9)	7 (0.7)	5 (2.1)	0 (0.0)	3 (2.1)	
Smoking						<0.05
Never smoker	1,120 (68.1)	703 (69.5)	152 (63.9)	163 (65.2)	102 (70.3)	
Quit	467 (28.4)	276 (27.3)	72 (30.3)	84 (33.6)	35 (24.1)	
Current smoker	49 (3.0)	27 (2.7)	11 (4.6)	3 (1.2)	8 (5.5)	
Missing	8 (0.5)	5 (0.5)	3 (1.3)	0 (0.0)	0 (0.0)	
Employment status						0.26
Non-employed/student	567(34.5)	365 (36.1)	84 (35.3)	76 (30.4)	42 (29.0)	
Work	1,023 (62.2)	615 (60.8)	143 (60.1)	168 (67.2)	97 (66.9)	
Missing	54 (3.3)	31 (3.1)	11 (4.6)	6 (2.4)	6 (4.1)	
Parity						0.06
First-time parents	717 (43.6)	420 (41.5)	110 (46.2)	126 (50.4)	61 (42.1)	
One child	590 (35.9)	367 (36.3)	92 (38.7)	80 (32.0)	51 (35.2)	
Two or more children	268 (16.3)	185 (18.3)	26 (10.9)	34 (13.6)	23 (15.9)	
Missing	69 (4.2)	39 (3.9)	10 (4.2)	10 (4.0)	10 (6.9)	
Nursery attendance						0.80
No	963 (58.6)	606 (59.9)	137 (57.6)	144 (57.6)	76 (52.4)	
At six months and one year	92 (5.6)	54 (5.3)	11 (4.6)	14 (5.6)	13 (9.0)	
At one year only	528 (32.1)	312 (30.9)	82 (34.5)	83 (33.2)	51 (35.2)	
At six months only	5 (0.3)	4 (0.4)	0 (0.0)	1 (0.4)	0 (0.0)	
Missing	56 (3.4)	35 (3.5)	8 (3.4)	8 (3.2)	5 (3.5)	
Breastfeeding at four months old						0.68
No	216 (13.1)	141 (14.0)	24 (10.1)	31 (12.4)	20 (13.8)	
Yes	1,402 (85.3)	853 (84.4)	212 (89.1)	215 (86.0)	122 (84.1)	
Missing	26 (1.6)	17 (1.7)	2 (0.8)	4 (1.6)	3 (2.1)	
Immunization completed						0.05
Yes	1,259 (76.6)	794 (78.5)	178 (74.8)	187 (74.8)	100 (69.0)	
No	385 (23.4)	217 (21.5)	60 (25.2)	63 (25.2)	45 (31.0)	

Participant characteristics are stratified according to pediatric vaccination facility use patterns during the routine childhood immunization period. Values are shown as number and percentage (%). Chi-square tests were used to examine group differences across the four facility use categories.

Table 3 shows the association between pediatric vaccination facility use patterns and routine childhood immunization completion as measured by crude and adjusted logistic regression analyses. Multiple facility use was associated with a higher odds of routine childhood immunization incompletion than single facility use. This association was the strongest for multiple facility use throughout the immunization period (adjusted odds ratio, 1.90; 95% confidence interval [CI], 1.24–2.91); however, multiple facility use at the first period (aOR, 1.41; 95% CI, 0.97–2.03) and multiple facility use at the second period (with this one being non-significant; aOR, 1.36; 95% CI, 0.95–1.95) showed trends toward a decreased odds of routine childhood immunization incompletion. Maternal age at delivery was not significantly associated with routine childhood immunization completion after adjustments. However, marital status emerged as a significant factor, with single mothers having higher odds of routine childhood immunization incompletion than married mothers (aOR, 2.54; 95% CI, 1.33–4.85).

**Table 3:** Logistic regression analysis of routine childhood immunization incompletion by pediatric vaccination facility use patterns

	**cOR**	**95% CI**	**aOR**	**95% CI**
Vaccination facility use patterns				
Single facility use throughout	ref		ref	
Multiple facility use at the first period	1.23	(0.89–1.71)	1.41	(0.97–2.03)
Multiple facility use in the second period	1.23	(0.89–1.70)	1.36	(0.95–1.95)
Multiple facilities used throughout	**1.65**	**(1.12–2.41)**	**1.90**	**(1.24–2.91)**
Maternal age at delivery (years)				
<20	0.52	(0.06–4.38)	0.68	(0.07–6.57)
20–24	0.79	(0.48–1.31)	0.87	(0.45–1.67)
25–29	0.82	(0.61–1.10)	1.02	(0.73–1.42)
30–34	ref		ref	
35–39	1.07	(0.80–1.43)	1.05	(0.76–1.45)
≥40	1.29	(0.75–2.22)	1.33	(0.72–2.43)
Educational level				
High school or less	ref		ref	
Vocational school/college	0.76	(0.58–1.00)	0.91	(0.66–1.25)
University or higher	**0.62**	**(0.45–0.85)**	0.87	(0.60–1.27)
Marital status				
Married	ref		ref	
Single	**1.86**	**(1.11–3.14)**	**2.54**	**(1.33–4.85)**
Smoking				
Never smoke	ref		ref	
Quit	**1.43**	**(1.11–1.83)**	1.29	(0.98–1.71)
Current smoker	**2.61**	**(1.45–4.70)**	1.58	(0.80–3.14)
Missing				
Employment status				
Non-employed or student	ref		ref	
Work	1.14	(0.89–1.46)	1.08	(0.78–1.49)
Parity				
First-time parent	ref		ref	
One child	**2.47**	**(1.87–3.27)**	**2.62**	**(1.93–3.56)**
Two or more children	**4.68**	**(3.39–6.46)**	**4.84**	**(3.37–6.96)**
Nursery attendance				
No	ref		ref	
At six months and one year	**1.93**	**(1.22–3.05)**	1.48	(0.87–2.52)
At six months only	1.21	(0.94–1.56)	1.08	(0.78–1.50)
At one year only	0.95	(0.11–8.53)	2.32	(0.19–27.72)
Breastfeeding at four months old				
No	ref			
Yes	0.77	(0.56–1.06)	0.82	(0.56–1.21)

Logistic regression analysis of factors associated with routine childhood immunization incompletion. Crude odds ratios (cORs) and adjusted odds ratios (aORs) with 95% confidence intervals (CIs) are presented. Adjusted models include all covariates of maternal age at delivery, educational level, marital status, smoking, employment status, parity, nursery attendance, and breastfeeding at four months of age. All results marked in bold indicate significant differences.

Maternal educational level and employment status were not significantly associated with routine childhood immunization completion in the adjusted model. While smoking status was significantly associated in the crude analysis, this association became non-significant after adjustment. Parity was strongly associated with routine childhood immunization completion: those with one child (aOR, 2.62; 95% CI, 1.93–3.56) and those with two or more children (aOR, 4.84; 95% CI, 3.37–6.96) had significantly higher odds of routine childhood vaccination incompletion than first-time parents. Nursery attendance and breastfeeding at four months old did not show significant associations with vaccination completion in the adjusted model.

## 4. Discussion

In this study, we investigated the association between pediatric vaccination facility use patterns and routine childhood immunization uptake/completion for a follow-up period of eight years. We found that the use of a single facility throughout the immunization period was superior to the use of multiple facilities and significantly associated with increased vaccination uptake, which in turn resulted in routine childhood immunization completion. To our knowledge, this is the first report in the literature on this issue using a large sample size enrolled at 15 JECS study areas in Japan. Among the total participants, 76.6% completed the vaccination schedule and 61.5% used a single clinic for vaccination. Regarding group analyses using a chi-square test, marital status, educational level, lower parity, never smoking, not attending nursery, and breastfeeding practice at four months old were significantly associated with complete childhood immunization. Adjusted logistic regression analysis further revealed that multiple facility use was more strongly associated with a higher odds of routine childhood immunization incompletion than single facility use. All these results collectively suggest that some of the participants’ particular background characteristics and single facility use throughout the immunization period can be valuable focus points for efforts at improving vaccination uptake and routine childhood immunization completion.

However, in addition to improving vaccination facilities and uptake, the effectiveness of vaccination strategies can vary across different sociodemographic groups and vaccine types. For instance, a study on influenza vaccine effectiveness in children under three years old in Japan found that vaccine effectiveness varied significantly between seasons and dosage schedules [19]. Future studies are thus needed to confirm the efficacy of routine vaccinations in children and their time-dependent efficacy in disease prevention. While our study focused on vaccination facility use patterns and their impact on vaccination uptake, broader strategies for improving vaccination rates must be considered. Previous research has highlighted the effectiveness of early vaccine education and informational interventions. For instance, studies have shown that providing vaccine education during the perinatal period can significantly improve parental knowledge and attitudes toward childhood vaccinations [20]. Additionally, the use of vaccine information booklets is an effective tool in increasing vaccine acceptance and completion rates among parents [21]. The MCHH may be fulfilling this role in Japan. Based on these findings and our results on vaccination facility use patterns, we propose that future research should explore integrated approaches combining targeted facility use strategies with early vaccine education and information dissemination. Such studies could examine how perinatal vaccine education programs and the distribution of informative materials at frequently used vaccination facilities may synergistically improve vaccination uptake and completion rates. This approach would address both the practical aspects of vaccine delivery and the educational needs of parents, potentially leading to comprehensive and effective vaccination promotion strategies.

Marital status emerged as a significant factor, with single mothers showing lower routine childhood immunization completion than married mothers. These findings are consistent with those of previous studies [22]. Furthermore, our findings on the association between multiple pediatric vaccination facility use patterns and immunization completion should be considered in the context of other socioeconomic factors, considering that a study using data from the JECS found that children of single-mother households were at higher risk of insufficient vaccination across all age groups studied [22]. The consistent association between single motherhood and lower vaccination rates across multiple studies, including our own, highlights the need for targeted interventions and support systems for single-parent households. Healthcare providers and policymakers should consider these socioeconomic vulnerabilities when designing strategies to improve vaccination coverage. By addressing the specific challenges faced by single mothers and other at-risk groups while optimizing vaccination facility use patterns, we can work toward more equitable and comprehensive immunization programs that protect all children, regardless of family circumstances.

The number of children in a family strongly influences vaccination uptake. First-time parenthood was associated with the highest rate of routine childhood immunization completion, followed by those with one child and those with two or more children. This finding aligns with those of previous studies, showing that vaccination rates tend to decrease as the number of children in a family increases [23]. These findings might be related to the busy lifestyle of mothers with multiple children, and they point toward the need to explain the importance and benefit of routine childhood immunization completion to mothers with multiple children and single mothers. This may help ensure that these mothers properly visit a pediatric facility and complete the vaccination schedule for their next child. Therefore, targeted communication strategies and support for first-time parents to address their unique concerns and information needs regarding childhood vaccination are needed. Healthcare providers should be prepared to engage in patient-centered conversations addressing the specific informational needs of first-time parents while respecting their beliefs and concerns [24].

Interestingly, factors such as maternal education level and employment status were not significantly associated with vaccination uptake after adjustments in the model. Hence, other factors may be more important in determining mothers’ vaccination behaviors, and these findings do not concur with those in the study by Forshaw et al. [25], where a higher maternal educational level was associated with higher child vaccination uptake. The odds of full childhood vaccination were 2.3 times higher for children whose mothers received secondary or higher education than for children whose mothers had no education [25]. The discrepancy between our study and this cited study suggests that the relationship between maternal education and vaccination uptake may be ascertained by differences in sample size, nationality, race, ethnicity, and education via mass media in addition to school education.

Our findings also suggest an association between facility switching patterns and immunization incompletion, but the directionality of this association warrants careful consideration. Families experiencing challenges in maintaining vaccination schedules—such as missed appointments, relocation, or dissatisfaction with services—might increase facility switching as a coping mechanism. This reverse causation dynamic, wherein incomplete immunization drives facility changes, has been observed in the context of other vaccine implementation challenges where access barriers precede behavioral adaptations [26]. Pediatricians’ diligent schedule management might not fully prevent this issue, as parents who miss critical vaccination windows could subsequently seek alternative facilities to compensate for delays. Our findings suggest that these families with documented facility switching patterns may require enhanced follow-up systems, such as centralized immunization registries or active recall programs, to mitigate subsequent dropout risks. Further research is needed to elucidate the causal mechanisms underlying this association.

A study on childhood vaccinations in Japan demonstrated a positive correlation between pediatrician density and vaccination rates for measles and DPT vaccines. It found that for every one-unit increase in the number of pediatricians per 10,000 children, the vaccination rate increased by 1.2% for measles and 1.9% for DPT vaccines [23]. These findings align with those of international studies that have also shown a relationship between healthcare provider availability and immunization coverage, suggesting that the supply of pediatricians plays a crucial role in promoting vaccination uptake and improving child health outcomes [23]. However, in Kochi Prefecture, where this study was conducted, while the number of pediatric facilities has not decreased significantly, there is a notable concentration of these facilities in the central area, particularly in Kochi City. The current situation is such that families have limited options when it comes to choosing environments for raising children. Given these circumstances in Kochi Prefecture, it becomes even more crucial to optimize the use of existing healthcare resources and develop strategies to maintain high vaccination rates despite the declining number of pediatric facilities. To address these challenges, local medical plans in Kochi Prefecture’s municipalities have implemented evidence-based strategies, such as provider reminder-recall systems and simultaneous vaccine administration. These approaches align with established methods for reducing missed vaccination opportunities [27, 28], demonstrating effectiveness even in regions with varying facility distributions.

### 4.1. Strengths

Our study has several strengths. First, to our knowledge, this was the first study report on the relationship between routine childhood vaccination uptake and pediatric facility use patterns applying a large sample size from 15 JECS study areas in Kochi Prefecture.

Second, we conducted this study in Kochi Prefecture, where the distribution of healthcare facilities is notably imbalanced despite the expansive geographical layout. Still, the Prefecture has demonstrated a commitment to creating an environment supportive of working parents, particularly mothers; in a recent nationwide survey, Kochi Prefecture ranked first among Japanese prefectures in providing a family-friendly environment for working mothers, considering factors such as the availability of public daycare services and private childcare options [29, 30]. These pieces of information highlight the importance of selecting Kochi Prefecture for our current study. The findings from Kochi Prefecture, known for its high rate of working women, can provide pioneering insights for other areas where opportunities for women’s social advancement are expected to increase in the future.

Third, we utilized a comprehensive approach to confirm children’s vaccination status validity, with pediatricians meticulously examining the MCHH to confirm both vaccination status and the facilities where the vaccinations were administered. This method ensured a high level of accuracy in data collection, as the MCHH serves as an official and detailed record of a child’s health and vaccination history in Japan [31]. By having pediatricians review these records, we were able to capture correct information about vaccination patterns and facility utilization that might not be apparent through other data collection methods.

### 4.2. Limitations

This study has some limitations. First, the data used were not necessarily collected at the same time points for all participants, which could produce variability. This limitation of data collection timing consistency is inherent to large-scale cohort studies that follow participants over extended periods. However, despite this limitation, the JECS is a large-scale cohort study that tracks subjects from pregnancy through to childhood, which is a design that allows for the collection of highly accurate and detailed data.

Second, the participation rate in this adjunct study was 23.8% of the eligible mothers from the JECS Kochi Regional Center, raising concerns about potential selection bias. It is possible that mothers who chose to participate in the adjunct study differed systematically from those who did not. For example, participants may have been more likely to engage in health-promoting behaviors such as maintaining a healthy diet or engaging in regular physical activity, may have had higher levels of education, or more stable household incomes [32, 33]. These factors may be associated with an overestimation of positive health behaviors or outcomes. While we observed that non-respondents had children with slightly longer device use at three years old, suggesting a potential underestimation of prolonged device use prevalence, we acknowledge that unmeasured confounders may still exist. Therefore, caution is warranted when generalizing our findings to the entire population of mothers in the JECS.

Third, complex life circumstances, such as the temporary relocation for purposes of childbirth (in Japanese, “*Satogaeri Shussan*”) or permanent relocation, may have been the reasons why the mothers had to visit multiple pediatricians, and these were not fully recorded in our study. These nuanced aspects of participants’ lives and healthcare-seeking behaviors could potentially influence vaccination patterns and diverse facility usage but were not captured in our analysis.

Fourth, the vaccination records in the MCHH alone do not provide information about disease history, but it is important to emphasize that the purpose of our study was different, and it did not focus on disease occurrence based on vaccination patterns.

Fifth, various potential confounding factors were not accounted for. While we included sociodemographic factors in our logistic regression model, we were unable to capture other important confounders such as child health status, parental attitudes toward vaccines, distance to healthcare facilities, and physician recommendation. These factors could influence both the choice of vaccination facility and uptake, potentially distorting the observed associations. For example, parents with concerns about vaccine safety may be more likely to seek multiple opinions or switch facilities, while those living far from healthcare facilities may face greater challenges regarding vaccination schedule adherence. Furthermore, our data did not capture the circumstances on the healthcare provider side, such as facility closures. Future studies should collect data on these potential confounders and employ more comprehensive analytical techniques to better understand the relationship between facility use and vaccination uptake.

Sixth, residual human error potential exists in the photo-based data transcription, given the methodology’s inherent dependence on manual record-keeping practices in MCHHs. The future implementation of maternal-child health apps is expected to digitize vaccination records, while the integration of electronic immunization data with clinical administration records through unified digital platforms will enable more precise and granular analyses, opening new possibilities for advancing this research field through access to standardized electronic health data systems.

## 5. Conclusions

Our study suggests a correlation between higher vaccination rates and the consistent use of a single pediatric clinic. Encouraging parents to utilize a single medical institution for their child’s vaccinations during the recommended period may be one approach to explore when seeking to improve infant vaccination coverage. This could lead to a more efficient vaccination schedule management and potentially decrease the occurrence of incomplete vaccinations.

## Abbreviations

aOR: adjusted odds ratio
CI: confidence interval
JECS: Japan Environment and Children’s Study
MCHH: Maternal and Child Health Handbook

## Supplementary information

**Additional file 1:** **Suppl. Figure 1.** Sample Image of the Maternal and Child Health Handbook. This file presents a sample photo of the Maternal and Child Health Handbook used in Japan. As shown in the image, for each type of vaccine, the date of administration is recorded along with the corresponding pediatrician’s stamp or signature and the name of the medical institution where the vaccination was performed.

## Declarations

### Availability of data and materials

It is not possible to share the raw research data publicly since data privacy could be compromised. However, the raw data are available from the corresponding author upon reasonable request, and any researcher interested in gaining access to the raw data can address their request to the corresponding author at the contact information mentioned in the manuscript. All data generated or analyzed during this study are included in this published article and its supplementary information.
