# Supplementary material for: Differences in routine childhood immunization uptake between single and multiple healthcare facility use: the Kochi Adjunct Study of Japan Environment and Children’s Study
Source: Environ Health Prev Med. 2025 Jun 28;30:51. doi: 10.1265/ehpm.25-00028 (PMC12206665; doi:10.1265/ehpm.25-00028)
Supplement: Supplementary file 1 — Additional file 1: Suppl. Figure 1. Sample Image of the Maternal and Child Health Handbook. This file presents a sample photo of the Maternal and Child Health Handbook used in Japan. As shown in the image, for each type of vaccine, the date of administration is recorded along with the corresponding pediatrician’s stamp or signature and the name of the medical institution where the vaccination was performed. [file ehpm-30-051-s001.pdf]

＊＊＊＊＊＊＊ 予防接種の記録 ＊＊＊＊＊＊＊

Immunization Record

BCG

接種年月日 Y/M/D (年齢)	ロット Lot.No.	接種者署名 Physician	備考 Remarks
平10. 12.10	KH 126	signature	

ジフテリア・百日せき・破傷風 Diphtheria · Pertussis · Tetanus

時期		ワクチンの種類 Vaccine	接種年月日 Y/M/D (年齢)	メーカー/ロット Manufacturer/ Lot.No.	接種者署名 Physician	備考 Remarks
第1期初回	1回	DPTワクチン	H.23.8.22	DPT 「タケダ」 Q034[illegible] EXP. 2012.10.1[illegible]	stamp	右上腕下1/3 0.5ml (S.C)
	2回	DPTワクチン	H.23.12.17	DPT 「タケダ」 Q034[illegible] EXP. 2012.10.1[illegible]	stamp	左上腕下1/3 0.5ml (S.C)
	3回	DPT +IPV	2015 10.13	化血研 DPT-IPV A025B Exp 2016.12.1	signature	[illegible]
第1期追加		DPT +IPV	2016 6.14	化血研 DPT-IPV A029C Exp 2017.3.16	signature	[illegible]

●薬剤などのアレルギー記入欄

不活化ポリオワクチン		接種年月日	メーカー/ロット	接種者署名	備考
初回接種	1回目 3~8wの間	25.1.21	イモバックス ポリオ J0016[illegible] サノフィパスツール	signature	
	2回目 3~8wの間	25.3.30	イモバックス ポリオ J0079 サノフィパスツール	signature	
	3回目				
追加接種	4回目				

麻しん(はしか) Measles ・ 風しん Rubella					
	第1期	24.6.8	MR 「タケダ」 Y139 EXP. 2013.1.29	signature	左[illegible]
	第2期	2016 5.17	MR 北里 HF061A Exp.16.10.14	signature	[illegible]

日本脳炎 Japanese Encephalitis

時期		接種年月日 Y/M/D (年齢)	メーカー/ロット Manufacturer/ Lot.No.	接種者署名 Physician	備考 Remarks
第1期初回	1回	2015 10.13	日脳 化血研 E056A Exp. 2017.2.19	signature	[illegible]5ml
	2回	2016 6.14	日脳 化血研 E062A Exp. 2018.2.25	signature	[illegible]5ml
第1期追加					

●単抗原で予防接種を受けた場合は、それが分かるように備考欄もしくはその他の予防接種欄に記入してください。
